# Radiomics Analysis Derived From LGE-MRI Predict Sudden Cardiac Death in Participants With Hypertrophic Cardiomyopathy

**DOI:** 10.3389/fcvm.2021.766287

**Published:** 2021-12-10

**Authors:** Jie Wang, Laura Bravo, Jinquan Zhang, Wen Liu, Ke Wan, Jiayu Sun, Yanjie Zhu, Yuchi Han, Georgios V. Gkoutos, Yucheng Chen

**Affiliations:** ^1^Department of Cardiology, West China Hospital, Sichuan University, Chengdu, China; ^2^College of Medical and Dental Sciences, Institute of Cancer and Genomic Sciences, University of Birmingham, Birmingham, United Kingdom; ^3^West China School of Public Health, Sichuan University, Chengdu, China; ^4^Department of Geriatrics, West China Hospital, Sichuan University, Chengdu, China; ^5^Department of Radiology, West China Hospital, Sichuan University, Chengdu, China; ^6^Paul C. Lauterbur Research Centre for Biomedical Imaging, Shenzhen Institutes of Advanced Technology, Chinese Academy of Sciences (CAS), Shenzhen, China; ^7^Department of Medicine (Cardiovascular Division), University of Pennsylvania, Philadelphia, PA, United States; ^8^Institute of Translational Medicine, University Hospitals Birmingham NHS Foundation Trust, Birmingham, United Kingdom; ^9^Health Data Research UK (HDR), Midlands Site, United Kingdom; ^10^Center of Rare Diseases, West China Hospital, Sichuan University, Chengdu, China

**Keywords:** hypertrophic cardiomyopathy, machine learning, sudden cardiac death, late gadolinium enhancement, radiomics

## Abstract

**Objectives:** To identify significant radiomics features derived from late gadolinium enhancement (LGE) images in participants with hypertrophic cardiomyopathy (HCM) and assess their prognostic value in predicting sudden cardiac death (SCD) endpoint.

**Method:** The 157 radiomic features of 379 sequential participants with HCM who underwent cardiovascular magnetic resonance imaging (MRI) were extracted. CoxNet (Least Absolute Shrinkage and Selection Operator (LASSO) and Elastic Net) and Random Forest models were applied to optimize feature selection for the SCD risk prediction and cross-validation was performed.

**Results:** During a median follow-up of 29 months (interquartile range, 20–42 months), 27 participants with HCM experienced SCD events. Cox analysis revealed that two selected features, local binary patterns (LBP) (19) (hazard ratio (HR), 1.028, 95% CI: 1.032–1.134; *P* = 0.001) and Moment (1) (HR, 1.212, 95%CI: 1.032–1.423; *P* = 0.02) provided significant prognostic value to predict the SCD endpoints after adjustment for the clinical risk predictors and late gadolinium enhancement. Furthermore, the univariately significant risk predictor was improved by the addition of the selected radiomics features, LBP (19) and Moment (1), to predict SCD events (*P* < 0.05).

**Conclusion:** The radiomics features of LBP (19) and Moment (1) extracted from LGE images, reflecting scar heterogeneity, have independent prognostic value in identifying high SCD risk patients with HCM.

## Introduction

Hypertrophic cardiomyopathy is an autosomal dominant genetic disease with a prevalence of 1:500 in the general adult population (1). Although most the patients with hypertrophic cardiomyopathy (HCM) have a good prognosis, it still is one of the primary reasons for sudden cardiac death (SCD) events (2). Therefore, early, precise stratification and further identification of patients with high risk, so as to provide appropriate preventive therapy in the form of an implantable cardioverter-defibrillator (ICD) placement, is important in clinical practice.

Previous studies reported the extent of late gadolinium enhancement (LGE) with more than or equal to 15% is associated with adverse outcomes in patients with HCM (3–5). However, HCM patients with a low amount of LGE may experience sudden cardiac death events. For example, 25 out of 37 events (6) and 28 out of 60 events (7) occurred in patients with HCM with an LGE extent (<15%). Thus, there remains a clinical need to identify novel LGE markers to improve risk stratification.

Since the performance of existing clinical models for predicting SCD in patients with HCM is, by and large, considered insufficient, there is potential value in employing radiomic models in conjunction with machine learning approaches as tools to further explore precise stratification (8, 9). Smole et al. reported that machine learning approaches have the potential of improving the accuracy in predicting ventricular tachycardia occurrence, heart failure, and ICD activation with AUCs of 0.9, 0.88, and 0.87, respectively (10). Cheng et al. reported three LGE-texture parameters that were significantly associated with composites of cardiovascular death in 67 patients with HCM with systolic dysfunction and (*P* < 0.05) (11). In addition, previous studies demonstrated that the location and patterns of LGE are associated with cardiovascular adverse events in cardiomyopathies (12, 13). For example, scar heterogeneity, quantified by entropy, has been used to assist appropriate ICD therapy decisions (12), and the combined presence of septal and free-wall LGE has been previously associated with SCD risk in patients with dilated cardiomyopathy (13). However, the prognostic value of LGE patterns in patients with HCM is unknown. Currently, LGE-radiomics features have been demonstrated to allow for the quantification of image features, as well as cater to potential pattern identification (11, 14, 15). The purpose of the current study was to identify significant radiomics features of LGE images in participants with HCM and assess their potential value in predicting SCD endpoint.

## Materials and Methods

### Study Population

The current study prospectively recruited 383 sequential participants with HCM, who underwent 3-T cardiovascular MRI imaging during the period between August 2012 and October 2018. Four participants were excluded from the study due to the poor quality of LGE images that were obtained for them. HCM was diagnosed based on the presence of an increased left ventricle (LV) wall thickness (≥15 mm), identified in one or more myocardial segments (or ≥13 mm in a first degree relative of an index patient with HCM), measured by echocardiography and cardiovascular MRI in the absence of secondary causes of hypertrophy (16). This study was approved by the Institutional Ethics Committee of West China Hospital, Sichuan University, and written informed consent was obtained from each participant. In addition, the study was registered with the Chinese clinical trial registry (URL: http://www.chictr.org.cn; Unique identifier: ChiCTR1900024094).

### Cardiovascular MRI Scans

Cardiovascular MRI gated by electrocardiogram was performed on a 3-T scanner (MAGNETOM Trio Tim, Siemens Healthcare Ltd., Erlangen, Germany) with a 32-channel cardiac phased-array receiver coil. Detailed MRI acquisition protocols are available in the Supplemental Methods section, which are also detailed in a previous report (17).

### Feature Extraction of LGE Images

A total of 157 quantitative features were extracted from all short-axis LGE-phase sensitive inversion recovery (PSIR) images for each participant and the process of image filtering and feature extraction was performed with MATLAB 2014 (Mathworks, Natick, MA, USA). The myocardium was initially manually delineated within the images and then the radiomic features were extracted from the segmented myocardium. These features were extracted from each slice of all the acquired LGE images separately and then they were combined. Detailed descriptions of each feature are provided in Supplementary Table 1. The feature extraction approach is consistent with a previously published work (18).

### Cardiovascular MRI Analyses

Left ventricle (LV) function and LGE measurement were analyzed using commercially available software (QMASS 8.1; Medis Medical Imaging Systems, Leiden, the Netherlands) by two experienced radiologists with 4 years of cardiovascular MRI experience. Both these radiologists were blinded from any clinical and other cardiovascular MRI information and data related to this study. All analysis methods were consistent with previous reports (17–19). LV maximal wall thickness (MWT) was defined as the greatest dimension anywhere within the LV myocardium. LGE was defined quantitatively by myocardial signal intensity of 6 *SD* from the normal myocardium (20, 21) and the amount of LGE was semi-automatically quantified using the QMASS 8.1 software (Medis Medical Imaging Systems, Leiden, the Netherlands) and manual adjustments were applied when necessary.

### Follow-Up

The start time of follow-up was defined as the time when the participants underwent cardiovascular MRI examination at baseline. The duration of follow-up was defined from the initial cardiovascular MRI visit until October 2019 or SCD (or aborted SCD) event. Follow-up data were retrieved from participants' medical records as well as telephone interviews. We documented the SCD endpoint, including the SCD event which was defined as a natural death caused by a cardiac cause characterized by a sudden loss of consciousness that occurs within 1 h after the onset of acute symptoms (22), as well as aborted SCD events consisting of ICD discharge due to ventricular tachycardia (VT) or fibrillation, and sustained VT.

### Feature Selection of LGE Images

**Figure 1:**
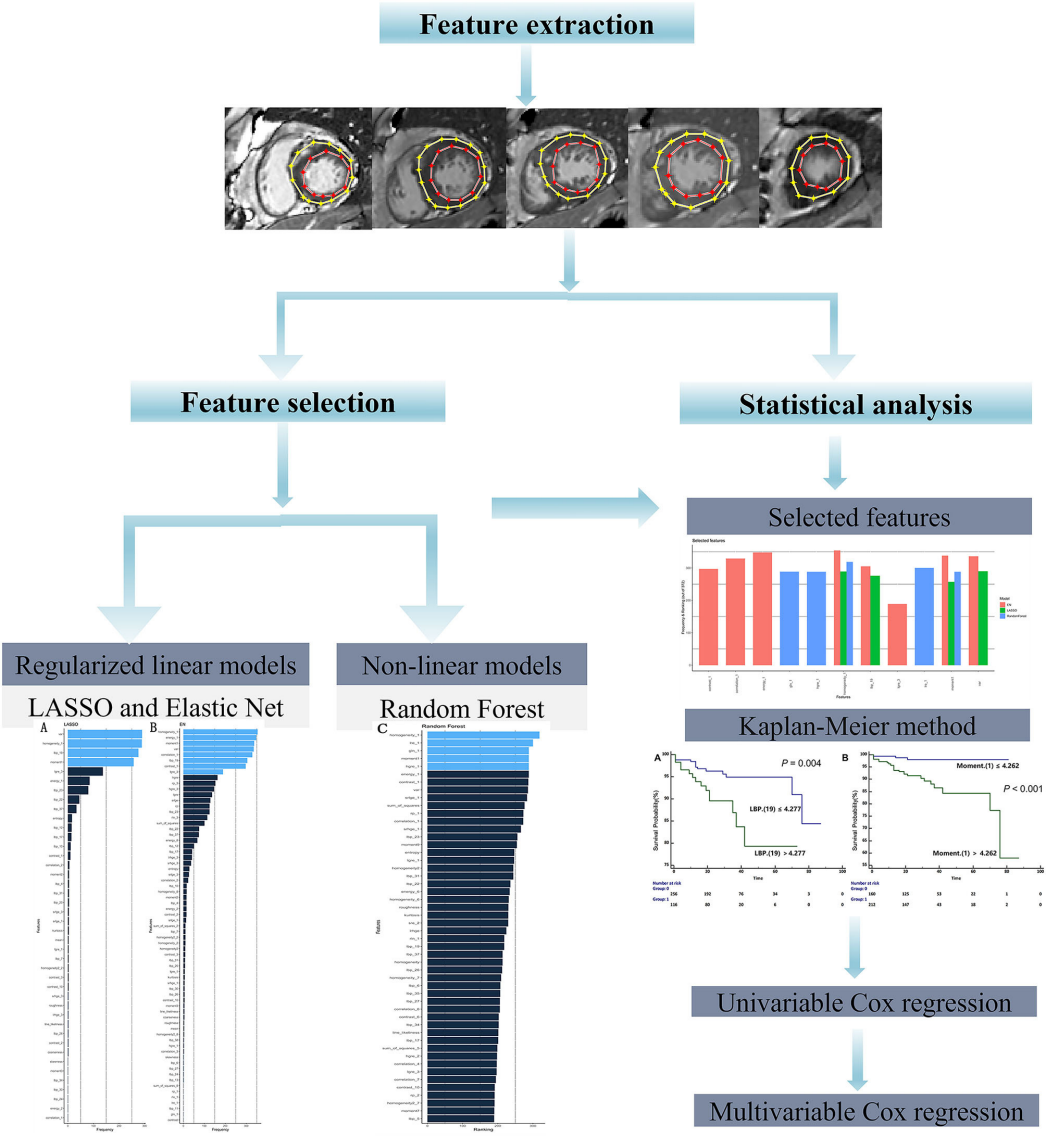
Radiomic workflow.

**Figure 2:**
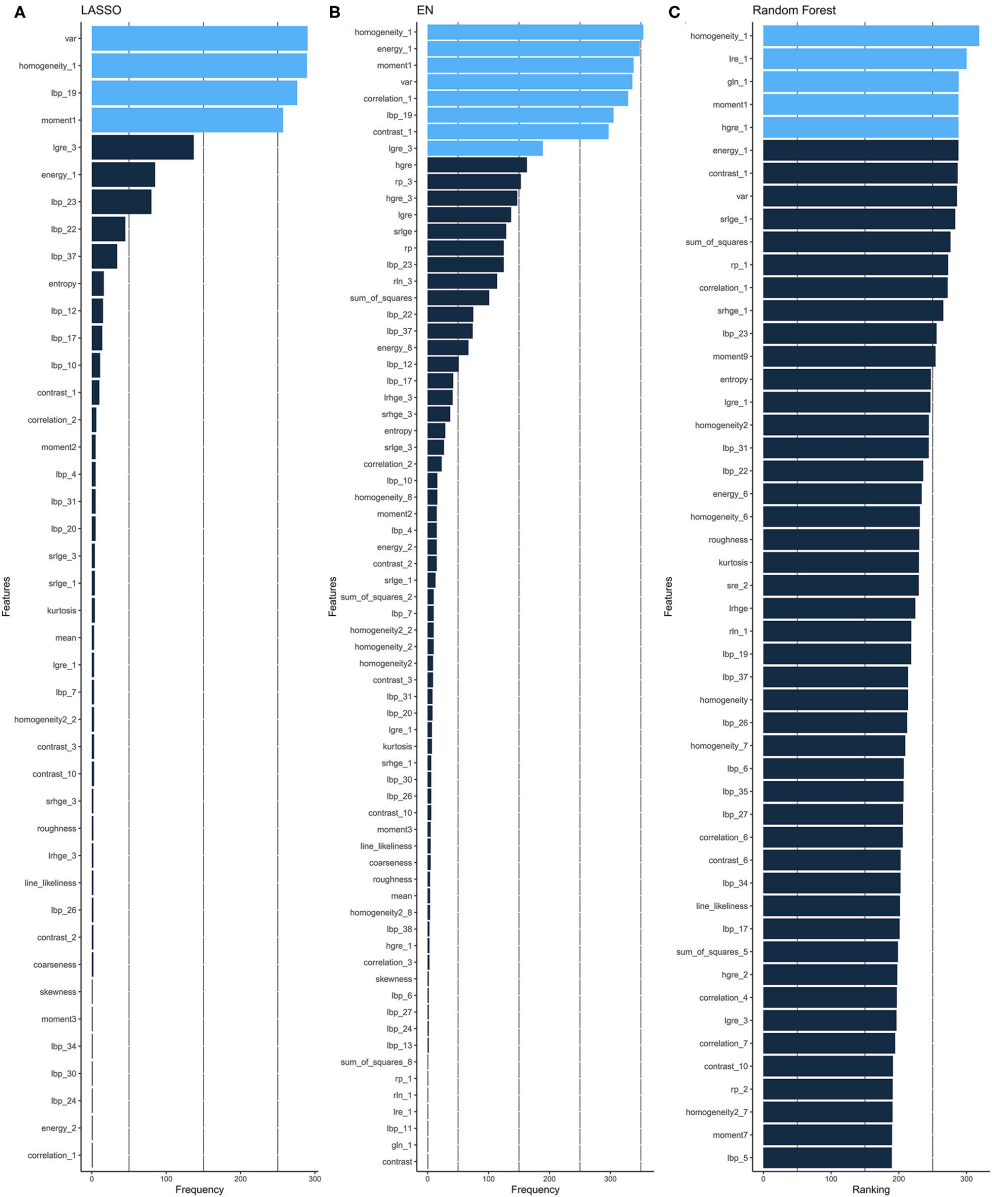
Feature selection. Ranking of important features of CoxNet with alpha = 1 i.e., LASSO **(A)** and alpha = 0.5 i.e., EN, **(B)** and Random Forest **(C)** algorithms. The features selected can be seen in light blue and are those that have appeared above a particular threshold of times. EN, elastic net; LASSO, Least Absolute Shrinkage and Selection Operator.

Given the high dimensionality of the dataset consisting of 379 patients and 157 different features, we performed feature selection through two different approaches: regularized linear model (CoxNet) (23) and non-linear model [Random Forest (RF)]. The radiomic workflow was shown in Figure 1. The CoxNet method fits the Cox proportional hazards regression model regularized by a specific penalty (23). We used two different penalty values (Figures 2A,B), alpha = 0.5 for elastic net (EN) behavior (24) where the penalty is less stringent and the correlated features were maintained, and alpha = 1 for a Least Absolute Shrinkage and Selection Operator (LASSO) behavior (25). The RF feature importance was determined based on a permutation method (26). In order to increase the robustness of our results, bootstrapping was performed, dividing the data into 400 different partitions. Additionally, 10-fold cross-validation for the optimum lambda was applied in each of the bootstrapped samples. The number of times a feature appears in a model, across all models generated by the different bootstraps, are reported and the features that appear more frequently across the models are assigned with a higher frequency value. Therefore, the chosen covariates correspond to those with the stronger and more robust effect across all linear and non-linear models. A detailed description is provided within the Supplemental Methods section. Figure 2 presents the top-ranking features across all different bootstraps.

**Figure 3:**
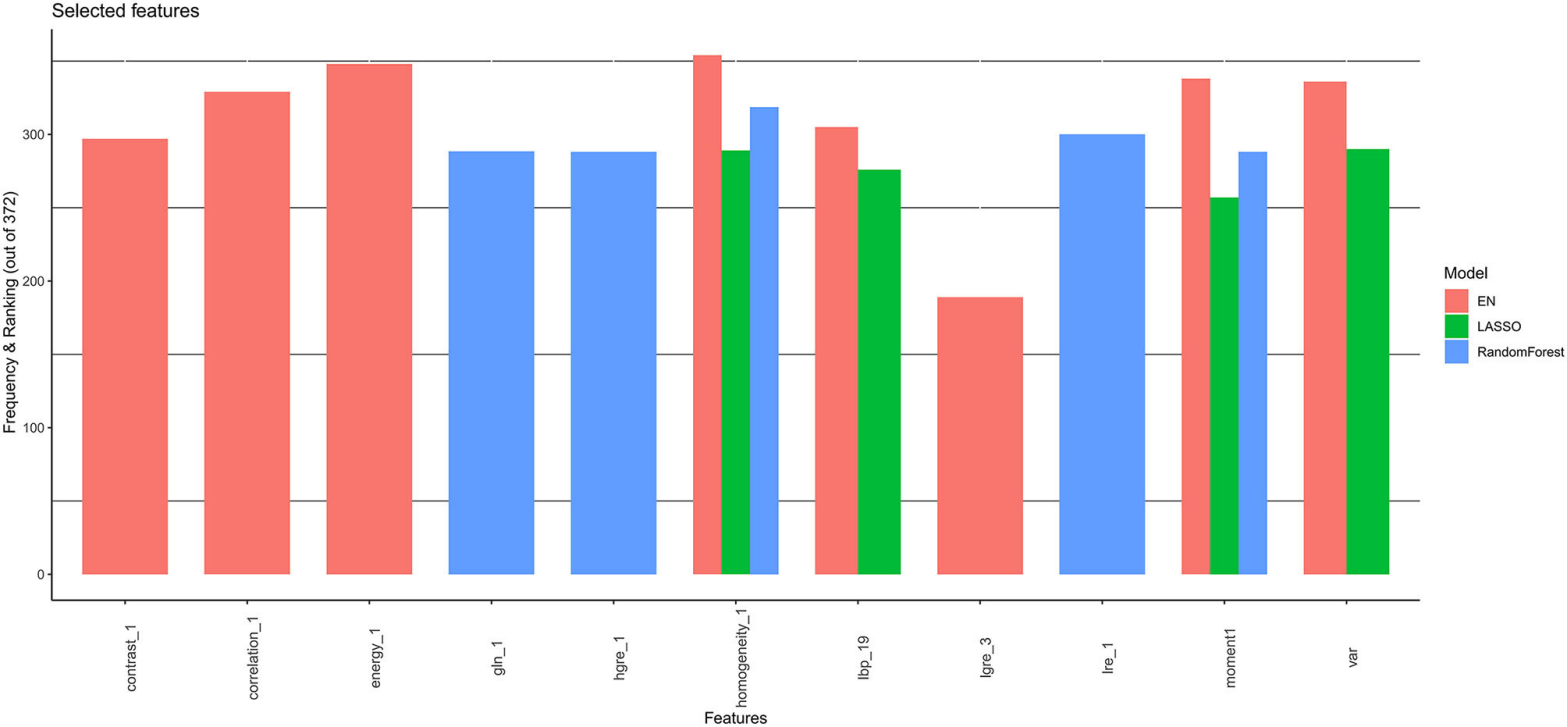
Final selected features by CoxNet and Random Forest models. EN, elastic net; LASSO, Least Absolute Shrinkage and Selection Operator.

Furthermore, all selected features were then combined at the end (Figure 3). Therefore, the covariates were chosen to correspond to those with the stronger and more robust effect on hazard across all linear and non-linear models. Additionally, the correlation between selected features was checked (Supplementary Figure 1) and the resulting uncorrelated ones were subsequentially chosen for the posterior survival analysis.

### Statistical Analyses

The CoxNet and Random Forest models were applied to select the most important radiomics features for the prediction of SCD endpoint and then 10-fold cross-validation was performed. The survival curves were established based on the Kaplan-Meier method, and comparisons were made using the log-rank test. The C-index was applied to evaluate the prognostic performance among different radiomics models. Univariable Cox regression analysis was used to evaluate the prognostic value of selected features or clinical variables for predicting the SCD endpoint. Factors with a *P*-value <0.10, identified in univariable analysis, were used for the multivariable Cox regression analysis, and a stepwise backward elimination was conducted to determine independent variables. So as to ensure the reproducibility of our radiomics analysis, the coefficient of variation (COV) and the intraclass correlation coefficient (ICC) were both reported. *P* < 0.05 values were considered statistically significant. All statistical analyses were performed using the R (version 3.5.2; The R Project for Statistical Computing, Birmingham, UK) and MedCalc (version 13; Ostend, Belgium) software.

## Results

### Demographic and Baseline Clinical Characteristics

Table 1 presents the baseline clinical and CMR characteristics of the HCM patients, as well as a comparison between participants with HCM reaching an SCD endpoint and ones without an SCD endpoint. Most participants were treated with β blockers (70%). There were no significant differences in the baseline clinical characteristics of sex, body mass index, body surface area, blood pressure, cardiac function, and MWT between the two subgroups (all *P* > 0.05). However, age and hypertension history, coronary artery disease (CAD), non-sustained VT (NSVT), and syncope showed significant differences in the two subgroups (Table 1). In addition, women were more likely to have an increased marker for SCD risk, based on European Society Cardiology HCM risk predictors, as well as being more likely to have a history of syncope and higher peak LVOT obstruction (Supplementary Table 2).

**Table 1:** Demographic and clinical characteristics in recruited participants with hypertrophic cardiomyopathy.

**Variable**	**HCM (*n* = 379)**	**Patients without SCD endpoint (*n* = 352)**	**Patients with SCD endpoint (*n* = 27)**	***P***
**Clinical data**				
Age (years)	48.6 ± 16.3	47.9 ± 16.3	58.5 ± 14.1	0.001^*^
Male gender, *n* (%)	203(54)	191(54)	12(44)	0.32
BMI (kg/m^2^)	23.7 ± 3.7	23.7 ± 3.6	23.9 ± 4.6	0.56
BSA (m^2^)	1.7 ± 0.2	1.7 ± 0.2	1.7 ± 0.2	0.65
SBP (mmHg)	123 ± 18	124 ± 18	121 ± 19	0.36
DBP (mmHg)	76 ± 12	75 ± 12	71 ± 13	0.1
HR (bpm)	73 ± 12	73 ± 11	74 ± 17	0.86
Diabetes mellitus, *n* (%)	26(7)	22(6)	4(15)	0.09
Hypertension, *n* (%)	92(24)	81(23)	11(41)	0.04^*^
CAD, *n* (%)	29(8)	24(7)	5(19)	0.03^*^
Peak LVOT obstruction, (mmHg)^†^	13.0 (5.0, 53.8)	11.0 (5.0, 55)	26.0 (5.0, 49.8)	0.55
NSVT, *n* (%)	59(16)	48(14)	11(41)	<0.001^*^
Family history of SCD, *n* (%)	50(13)	44(13)	6(22)	0.15
History of Syncope, *n* (%)	70(19)	57(16)	13(48)	<0.001^*^
**Cardiac medications**				
β blocker, *n* (%)	265(70)	243(69)	22(82)	0.17
**CMR data**				
LVEF (%)	62.6 ± 10.2	63.0 ± 9.5	59.0 ± 14.3	0.25
LVEDVi (mL/ m^2^)	82.0 ± 21.9	80.9 ± 18.2	95.7 ± 48.1	0.19
LVESVi (mL/ m^2^)	31.5 ± 16.7	30.5 ± 13.2	43.5 ± 39.6	0.23
RVEF (%)	60.9 ± 9.2	61.0 ± 9.2	59.3 ± 9.3	0.25
RVEDVi (mL/ m^2^)	65.3 ± 15.5	65.6 ± 15.5	61.8 ± 15.4	0.12
RVESVi (mL/ m^2^)	25.5 ± 8.9	25.6 ± 9.0	24.7 ± 6.8	0.85
LA size (mm)	40.2 ± 7.5	40.0 ± 7.4	43.2 ± 8.3	0.05
Maximum LV wall thickness (mm)	22.5 ± 5.7	22.6 ± 5.7	21.6 ± 6.1	0.43
LV Massi (g/m^2^)	99.4 ± 36.5	98.8 ± 35.2	107.6 ± 50.3	0.90
LGE%	8.6 ± 8.8	7.8 ± 7.9	14.4 ± 11.7	0.001^*^

* *P < 0.05*;

*HCM, hypertrophic cardiomyopathy; BMI, body mass index; BSA, body surface area; SBP, systolic blood pressure; DBP, diastolic blood pressure; HR, heart rate; LV, left ventricle; RV, right ventricle; EF, ejection fraction; SCD, sudden cardiac death; CAD, coronary artery disease; NSVT, non-sustained ventricular tachycardia; LOVT, left ventricular outflow tract gradient; EDVI, end-diastolic volume index; ESVI, end-systolic volume index; LV massi, LV mass index; LGE, late gadolinium enhancement*.

† *Obstructive HCM was defined as LV outflow tract gradient ≥30 mmHg at rest at echocardiography*.

*SCD, sudden cardiac death LBP, Local binary patterns; HGRE, high gray-level run emphasis; GLN, gray-level non-uniformity; LGRE, low gray-level run emphasis; LRE, long-run emphasis*.

Furthermore, there was a significant difference in LGE burden, when quantified as LGE% (14.4 ± 11.7 % vs. 7.8 ± 7.9 %; *P* =0.001), in participants with HCM with an SCD endpoint compared to patients without an SCD endpoint. Overall, participants with HCM who reached an SCD endpoint were had higher incidences of hypertension, CAD, syncope, NSVT, and higher LGE% (all *P* < 0.05, Table 1).

### Outcomes

During a follow-up period of 29 months (interquartile range [IQR] 20–42 months), 27 (7%) participants reached an SCD endpoint, including 10 (37%) participants that experienced SCD, 17 participants (63%) experienced aborted SCD events, including 9 patients with appropriate ICD discharge events due to VT or ventricular fibrillation (VF), and 8 patients sustained VTs.

### Features Extraction and Performance of Selected Features in Predicting SCD Endpoint

The application of CoxNet and Random Forest resulted in the selection of eleven, out of 157 radiomics features, namely local binary patterns (LBP) (19), moment (1), variance, high gray-level run emphasis (HGRE) (1), low gray-level run emphasis (LGRE) (3), long-run emphasis (LRE) (1), homogeneity (1), energy (1), contrast (1), gray-level non-uniformity (GLN) (1) and correlation (1) (Figure 3). The correlations among the 11 features are presented in Supplementary Figure 1. LBP (19) is the only feature that is not statistically associated with other the 10 radiomics features.

### Survival Analysis

**Figure 4:**
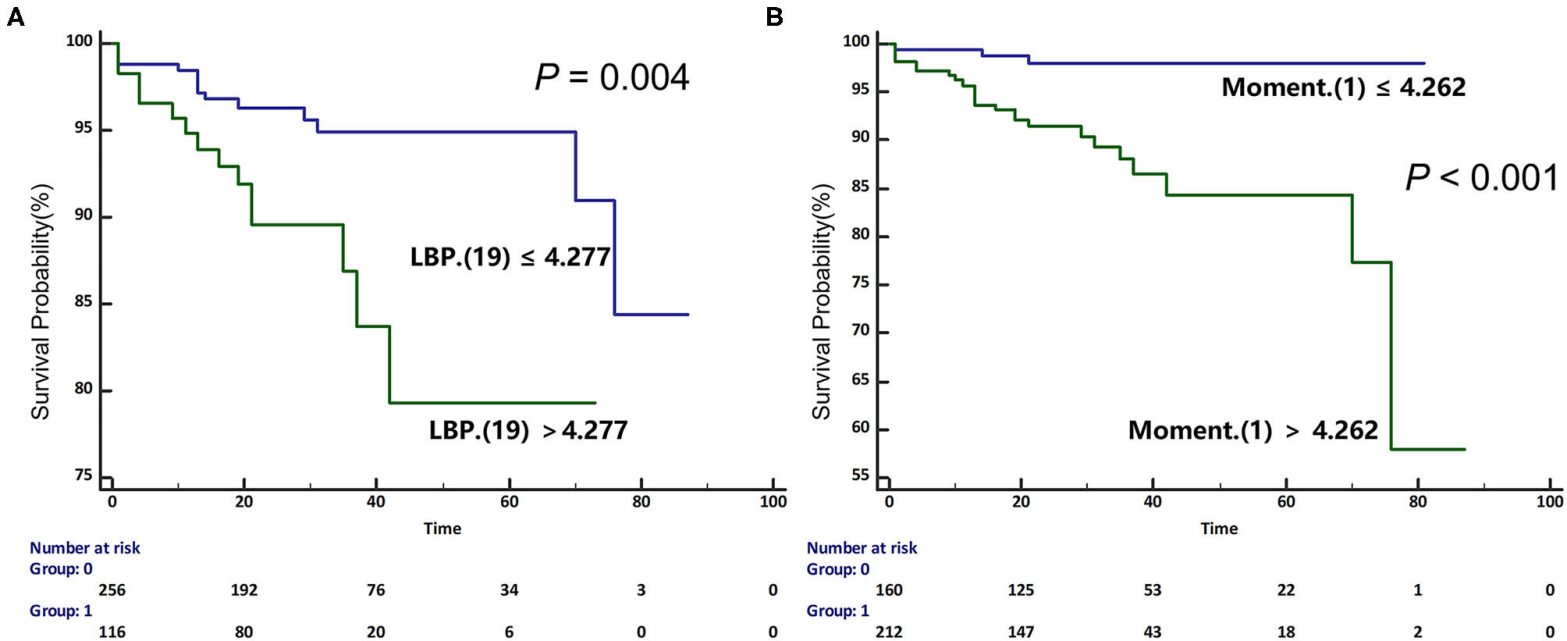
Kaplan-Meier (KM) curves for predicting sudden cardiac death endpoint in participants with HCM based on the median values of selected radiomics features [**A**. Local binary patterns (LBP) (19); **B**. Moment (1)]. HCM, hypertrophic cardiomyopathy; SCD, sudden cardiac death.

The univariable Cox regression analysis revealed that age, CAD, NSVT, syncope history, LVEDVi, LA size, and LGE% were significantly associated with the SCD endpoint (*P* < 0.05, Table 2). In addition, each of the 11 features had a significant prognostic value for predicting SCD endpoints (all *P* < 0.05, Table 2). In the multivariable Cox analysis, the age [HR: 1.051 (1.021, 1.082); *P* = 0.001], NSVT [HR: 2.763 (1.258, 6.067); *P* = 0.012], history of syncope [HR: 3.761 (1.688, 8.379); *P* = 0.001], LBP(19) [HR: 1.212 (1.032, 1.423); *P* = 0.02] and Moment (1) [HR: 1.082 (1.032, 1.134); *P* = 0.001] remained as independent predictors for SCD endpoints (Table 2). The HCM patients, with higher LBP (19) of (≥4.277 cutoff value), exhibited a significantly higher rate of reaching the SCD endpoint (*P* = 0.004; Figure 4A). In addition, higher moment (1) (≥4.262) also exhibited a significantly higher rate of reaching the SCD endpoint (*P* < 0.001, Figure 4B). To further assess the predictive power of the selected radiomics features to predict SCD endpoint, we first fitted a Cox regression model to the univariately significant risk predictors (age, CAD, NSVT, syncope history, LVEDVi, LA size, and LGE%). The C index of the risk model for predicting SCD endpoints is 0.834 ± 0.041. The risk model was slightly improved by the addition of the selected radiomics features (LBP 19 and Moment 1) with a C index of 0.852 ± 0.038. In addition, the univariately significant risk predictor was improved by the addition of the selected radiomics features, namely LBP (19) and Moment (1), to predict SCD events (Table 3).

**Table 2:** Univariable and multivariable Cox regression analysis of clinical variables and selected radiomics features for predicting the sudden cardiac death endpoint in participants with HCM.

**Variables**	**Univariate analysis**	***P***	**Multivariate analysis**	***P***
	**HR (95% CI)**		**HR (95% CI)**	
Sex, *n* (%)	1.441(0.677, 3.069)	0.346		
Age (years)	1.047(1.018, 1.076)	**0.001** ^*^	1.051(1.021,1.082)	0.001^*^
Height (cm)	0.997(0.966, 1.029)	0.854		
Weight (kg)	1.002(0.972, 1.032)	0.925		
BMI (kg/m^2^)	1.020(0.920, 1.131)	0.708		
BSA (m^2^)	0.885(0.149, 5.261)	0.885		
SBP (mmHg)	0.990(0.968, 1.012)	0.384		
DBP (mmHg)	0.091(0.936, 1.005)	**0.085**		
HR (bpm)	1.005(0.974, 1.038)	0.746		
Diabetes mellitus, *n* (%)	2.157(0.744, 6.260)	0.159		
Hypertension, *n* (%)	1.927(0.896, 4.146)	0.104		
CAD, *n* (%)	2.739(1.040, 7.217)	0.043^*^		
Peak LVOT obstruction, (mmHg)	1.002(0.991, 1.013)	0.728		
NSVT, *n* (%)	4.019(1.885, 8.569)	**0.001** ^*^	2.763(1.258, 6.067)	0.012^*^
Family history of SCD, *n* (%)	2.118(0.853, 5.256)	0.108		
History of Syncope, *n* (%)	4.309(2.032, 9.137)	**<0.001** ^*^	3.761 (1.688, 8.379)	0.001^*^
**CMR parameters**				
LVEF (%)	0.972(0.941, 1.004)	**0.082**		
LVEDVi (mL/m^2^)	1.018(1.009, 1.028)	<0.001^*^		
LVESVi (mL/m^2^)	1.023(1.013, 1.034)	**<0.001**		
RVEF (%)	0.985(0.948, 1.024)	0.442		
RVEDVi (mL/m^2^)	0.988(0.964, 1.012)	0.330		
RVESVi (mL/m^2^)	0.990(0.947, 1.035)	0.662		
LA size (mm)	1.059(1.006, 1.113)	**0.029** ^*^		
Maximum LV wall thickness (mm)	0.964(0.895, 1.038)	0.335		
LV Massi (g/m^2^)	1.005(0.996, 1.015)	0.301		
LGE %	1.065(1.033, 1.099)	**<0.001** ^*^		
Contrast (1)	1.164(1.094,1.239)	**<0.001** ^*^		
Variance	2.208(1.562,3.122)	**<0.001** ^*^		
Energy (1)	3.482(2.112,5.741)	**<0.001** ^*^		
HGRE (1)	1.461(1.145,1.863)	**0.002** ^*^		
LGRE (3)	1.598(1.206,2.116)	**0.001** ^*^		
LRE (1)	1.585(1.195,2.102)	**0.003** ^*^		
GLN (1)	1.187(1.103,1.277)	**<0.001** ^*^		
Homogeneity (1)	3.115(1.978,4.907)	**<0.001** ^*^		
Correlation (1)	1.703(1.273,3.378)	**<0.001** ^*^		
Moment (1)	1.087(1.050,1.126)	**<0.001** ^*^	1.082(1.032,1.134)	0.001^*^
LBP (19)	1.332(1.125,1.576)	**0.003** ^*^	1.212(1.032, 1.423)	0.020^*^

* *P < 0.05*;

*The bold values are less than 0.1; abbreviation as Table 1*.

**Table 3:** Comparisons of models including univariately significant risk predictors and selected radiomics features for SCD prediction by C statistic in participants with HCM.

Models^*^	**C statistic**	**Estimated variance**
Age	0.686	0.054
Age + LBP. (19) + Moment. (1)	0.815	0.039
CAD	0.547	0.039
CAD + LBP. (19) + Moment. (1)	0.745	0.051
NSVT	0.626	0.05
NSVT + LBP. (19) + Moment. (1)	0.743	0.056
syncope history	0.663	0.051
syncope history + LBP. (19) + Moment. (1)	0.777	0.051
LVEDVi	0.572	0.072
LVEDVi + LBP. (19) + Moment. (1)	0.703	0.066
LA size	0.612	0.061
LA size + LBP. (19) + Moment. (1)	0.702	0.067
LGE%	0.688	0.057
LGE% + LBP. (19) + Moment. (1)	0.724	0.059

* *Abbreviation as Table 1*.

### Reproducibility of Feature Extraction

A very good intra- and inter-observer reproducibility of feature extraction [inter-observer intraclass correlation coefficient: 0.92(0.89–0.98); intra-observer ICC:0.91(0.87–0.96)] was identified.

## Discussion

We reported that radiomics features, namely LBP (19) and Moment (1), derived from LGE images have independently and significantly predictive value of SCD endpoint in patients with HCM. Furthermore, the univariately significant clinical and imaging risk predictor was improved by the addition of the selected radiomics features.

Previous studies have confirmed the relationship between the extent of LGE and life-threatening ventricular arrhythmias in HCM patients (3). In addition, a recent study by Freitas et al. (493 HCM patients; 23 SCD or aborted SCD events), reported that the amount of LGE as a predictor (C-statistic 0.84; 95% CI: 0.76–0.91) outperformed the AHA or the ESC risk model in identifying patients with HCM at high SCD risk (4). Moreover, the latest enhanced AHA model, proposed by Maron et al., demonstrated that the ESC risk model could get significant improvement when adding LGE% with a cut-off value greater or equal to 15% (27). However, patients with HCM with the LGE extent (<15%) may experience high-risk cardiovascular events due to the diverse HCM phenotypic spectrum (5, 6). This may be partially explained by the different fibrosis patterns (28). The heterogeneity of fibrosis could not only affect electric conduction barriers but also facilitate the formation of critical isthmuses of viable myocytes which support re-entrant circuits and further cause arrhythmogenic cardiomyopathy (29). Furthermore, a previous study reported that spatial heterogeneity of fibrosis correlates directly with ventricular arrhythmia risk and the degree of risk would be higher when the spatial size and degree of fibrotic heterogeneity are increased (11, 15, 30). Therefore, LGE heterogeneity may play an important role in predicting SCD events necessitating the exploration of its prognostic value, the identification of potential markers, and the improvement of the characterization and stratification of LGE in patients with HCM. Our present study identified that radiomics features from LGE images, representing different patterns of myocardial fibrosis, have prognostic value for the identification of high-risk patients with HCM.

Radiomics forms a fairly recent image analysis technique that could be employed to describe the various distribution and patterns of signal intensity (SI) within segmented regions of interest (ROI). It provides a standardized formula for the quantification of intensity, shape, and texture features in medical images (31). Therefore, radiomics analysis, by identifying these unique SI patterns, may improve diagnosis (32, 33) and prognostic accuracy (34). Neisius et al. reported that the selected texture features from T1 mapping images could help to differentiate HCM from hypertension patients with the c-statistic of 0.89 (95% CI:0.77–1) (32). In addition, Baeßler et al. reported texture features from non-contrast T1-weighted images to have excellent accuracy to discriminate between HCM patients and healthy controls (sensitivity: 91%; specificity: 93%) (33). However, the prognostic value of radiomics features derived from T1 images is unknown. Kotu et al. recruited 34 individuals with chronic myocardial infarction and reported the texture features extracted from LGE images, including size, location, and heterogeneity of the scar, showed the excellent discriminative ability to detect high-risk arrhythmic patients with post-myocardial infarction (34). In a study of 23 patients with HCM, Amano et al. also identified significant differences in LGE texture features between patients with HCM with a history of ventricular tachyarrhythmias and those with not, which suggested that LGE texture analysis could provide information in patients with HCM with ventricular tachyarrhythmias (28). Our study further found that radiomics features from LGE-MRI have independent prognostic value for the identification of high SCD risk patients with HCM.

Cheng et al. included 67 patients with HCM with systolic dysfunction and reported that three texture parameters, namely X0_H_skewness (HR = 0.783, CI: 0.691–0.889), X0_GLCM_cluster_tendency (HR = 0.735, CI: 0.616–0.877) and X0_GLRLM_energy (HR = 1.344, CI: 1.173–1.540), were significantly associated with composites of cardiovascular death (*P* < 0.05) (11). The following points differentiate the work presented here from this work. First, Cheng et al. recruited a small HCM cohort (67 HCM patients) and based the validation of the significant prognostic value of three features on a univariable Cox analysis. Following a multivariable Cox analysis, and after adjusting for other clinical variables, these three features did not retain a statistically similar prognostic performance. Furthermore, the endpoints in that study were defined as composites of cardiovascular deaths events, including cardiac death due to progression of heart failure and unscheduled heart failure hospitalization. In our study, not only did we validate the independent prognostic value of radiomics features to identify specific SCD events in a larger cohort, but also, we were able to confirm that the combination of different, independent radiomic features resulted in increased performance, demonstrating that their inclusion into clinical models has incremental value.

LBP represented a rotation-invariant image descriptor, computed from discrete Fourier transforms of LBP histograms, and high LBP values reflect increased pixel-level heterogeneity in the myocardium scar (35). The feature of the moment was acquired through the construction of a gray-level co-occurrence matrix (GLCM) and reflected the local homogeneity from an image (31). These features aim to quantify the heterogeneity and complexity of the SI matrix. Therefore, these important, selected LGE-MRI features may reflect the myocardial fibrotic heterogeneity, and our study highlighted their importance in the prognostic evaluation of HCM patients and may provide a new sight into myocardial damage characterization. Furthermore, more prospective studies are needed to assess whether radiomics features, reflecting the heterogeneity of fibrosis, could be the main criterion of ICD treatment potentially aiding and igniting the further studies of the pathological mechanism of myocardial fibrosis in patients with HCM.

Our study employed a radiomics based approach to explore its prognostic value for identifying SCD endpoint in patients with HCM and demonstrated that radiomics features significantly improve our ability to stratify these patients. Our findings suggest that the extraction of radiomics features could potentially entail a new approach that caters to the exploration of individualized precise risk stratification in patients with HCM.

## Study Limitations

First, it is a single-center study, with all participants residing within the same geographical region of China. Therefore, external validation carried across other centers at different geographical regions is necessary. Moreover, our study included modest sample size, and it is important to validate the findings across a larger HCM population.

## Conclusion

In the present study population, we found that LV radiomics features extracted from LGE images, reflecting myocardial fibrosis heterogeneity, can provide an independent prognostic factor for SCD events, as well as helping to improve risk stratification in patients with HCM.

## Data Availability Statement

The raw data supporting the conclusions of this article will be made available by the authors, without undue reservation.

## Ethics Statement

The studies involving human participants were reviewed and approved by the Institutional Ethics Committee of West China Hospital, Sichuan University. The patients/participants provided their written informed consent to participate in this study. Written informed consent was obtained from the individual(s) for the publication of any potentially identifiable images or data included in this article.

## Author Contributions

YC, GG, and YH: conceptualization and project administration. JW and LB: methodology and writing—original draft preparation. JW, LB, and KW: formal analysis. JW, KW, JZ, and WL: investigation. JW, LB, KW, YZ, JZ, and WL: data curation. YC, GG, JS, YZ, and YH: writing—review and editing. YC, GG, JS, and YH: supervision. YC: funding acquisition. All authors contributed to the article and approved the submitted version.

## Funding

JW acknowledges support from China Scholarship Council (201906240180). LB acknowledges support from a Wellcome Trust 4-year studentship program in mechanisms of inflammatory disease (MIDAS: 215182/Z/19/Z). GG acknowledges support from the NIHR Birmingham ECMC, the NIHR Birmingham SRMRC, Nanocommons H2020-EU (731032), MAESTRIA (Grant Agreement ID 965286), and the MRC Health Data Research UK (HDRUK/CFC/01), an initiative funded by UK Research and Innovation, Department of Health and Social Care (England) and the devolved administrations, and leading medical research charities. This work was supported by a grant from 1·3·5 projects for disciplines of excellence, West China Hospital, Sichuan University (ZYJC18013).

## Author Disclaimer

The views expressed in this publication are those of the authors and not necessarily those of the NHS, the National Institute for Health Research, the Medical Research Council, or the Department of Health.

## Conflict of Interest

The authors declare that the research was conducted in the absence of any commercial or financial relationships that could be construed as a potential conflict of interest.

## Publisher's Note

All claims expressed in this article are solely those of the authors and do not necessarily represent those of their affiliated organizations, or those of the publisher, the editors and the reviewers. Any product that may be evaluated in this article, or claim that may be made by its manufacturer, is not guaranteed or endorsed by the publisher.
