# Supplementary material for: Radiomics Analysis Derived From LGE-MRI Predict Sudden Cardiac Death in Participants With Hypertrophic Cardiomyopathy
Source: Front Cardiovasc Med. 2021 Dec 10;8:766287. doi: 10.3389/fcvm.2021.766287 (PMC8702805; doi:10.3389/fcvm.2021.766287)
Supplement: Supplementary file 1 [file Data_Sheet_1.PDF]

Supplementary Material

1 Supplementary Method

1.1 Cardiovascular MRI Scans

Balanced steady-state free precession (bSSFP) cine images of the entire LV, from the base to the apex in consecutive short-axis views, were acquired during breath-holds. The inversion time was individually optimized to null normal myocardial signal using an inversion time scout sequence. For the steady-state free precession cine images of the entire LV, the scan parameters were as follows: repetition time, 3.4 ms; echo time, 1.3 ms; temporal resolution, 42 ms; flip angle, 50°; field of view, 320–340 mm; and matrix size, 256 × 144. The reconstructed in-plane spatial resolution was 1.4 × 1.3 mm, and the slice thickness was 8 mm with no gap. Late gadolinium enhancement (LGE) images were acquired 10–15 minutes after intravenous administration of 0.15 mmol/kg of gadopentetate dimeglumine (Gd-DTPA, Magnevist, Bayer Healthcare), using a phase-sensitive inversion recovery sequence with short axis views. The LGE sequence parameters with steady state free precession were as follows: the time to acquire a single line of k-space = 6.6 ms; the number of k-space lines acquired in one cardiac cycle= 16; echo time = 1.56 ms; flip angle = 20°; matrix size = 256 × 192; FOV = 340 × 320 mm2; spatial resolution = 1.5 × 1.3 mm2; slice thickness = 8 mm.

1.2 The assessment of LGE image quality

The radiologists respectively scored the image quality following the criteria listed below: 1. Four points: the image is clear, no artifacts, and the enhanced and non-enhanced areas are clearly displayed; 2. Three points: the image has a little artifact but can be distinguished between enhanced and non-enhanced areas; 3. Two points: image artifacts are obvious, LGE area is suspicious and cannot be

clearly determined; 4. One point: the image artifact is serious, and it is impossible to determine the LGE and non-LGE areas. LGE images with a score of 1 or 2 were deemed poor and were excluded.

1.3 Feature Selection of LGE Images

We performed feature selection by applying regularized cox regression and random forest in 400 different bootstraps of our dataset (400 different training and testing partitions (85:15)). Models were fitted for all these partitions, and the most important features were assessed. Features were selected based on being most important across all the features above the means of quantiles 5 (maximum number) and 2 (the 25th percentile or lower quartile) for both of the regularization algorithms and the top 5 important variables for random forest). The cross validation is applied for the tuning of the hyperparameter lambda required for the regularization algorithms, where the minimum lambda is selected after a 10-fold cross validation. Therefore, the selected features of the regularization algorithm are the result of the application of 400 different models, each tuned through a 10-fold cross validation. All packages and code used is available at https://github.com/InFlamUOB/RadiologyCox.

The packages of the regularized linear (CoxNet) and non-linear model (Random Forest) packages were “ *ranger*”, a fast implementation of Random Forests for high dimensional data in C++ and R [1] , “*survival* ”, *a package for survival analysis in R* [2], and “ *glmnet* ” [3]. The detailed methodology can be found in the code made available at (https://github.com/InFlamUOB/RadiologyCox)

1.4 Reproducibility of feature extraction

Reproducibility of feature extraction was tested on a random sample of 50 patients with HCM. To assess intra-observer variability, the same observer (J.W., with 4 years of cardiovascular MRI experience) who was blinded to clinical and other cardiovascular MRI data, delineated manually on the images twice and the radiomic features were extracted from the segmented myocardium in the selected sample twice, with an interval of at least 1 month between feature extraction. To assess

interobserver variability, another observer (K.W.; with 4 years of cardiovascular MRI experience) conducted the same analysis once and was blinded to the first observer's results of the same sample.

2 Supplementary Figures and Tables

2.1 Supplementary Figures

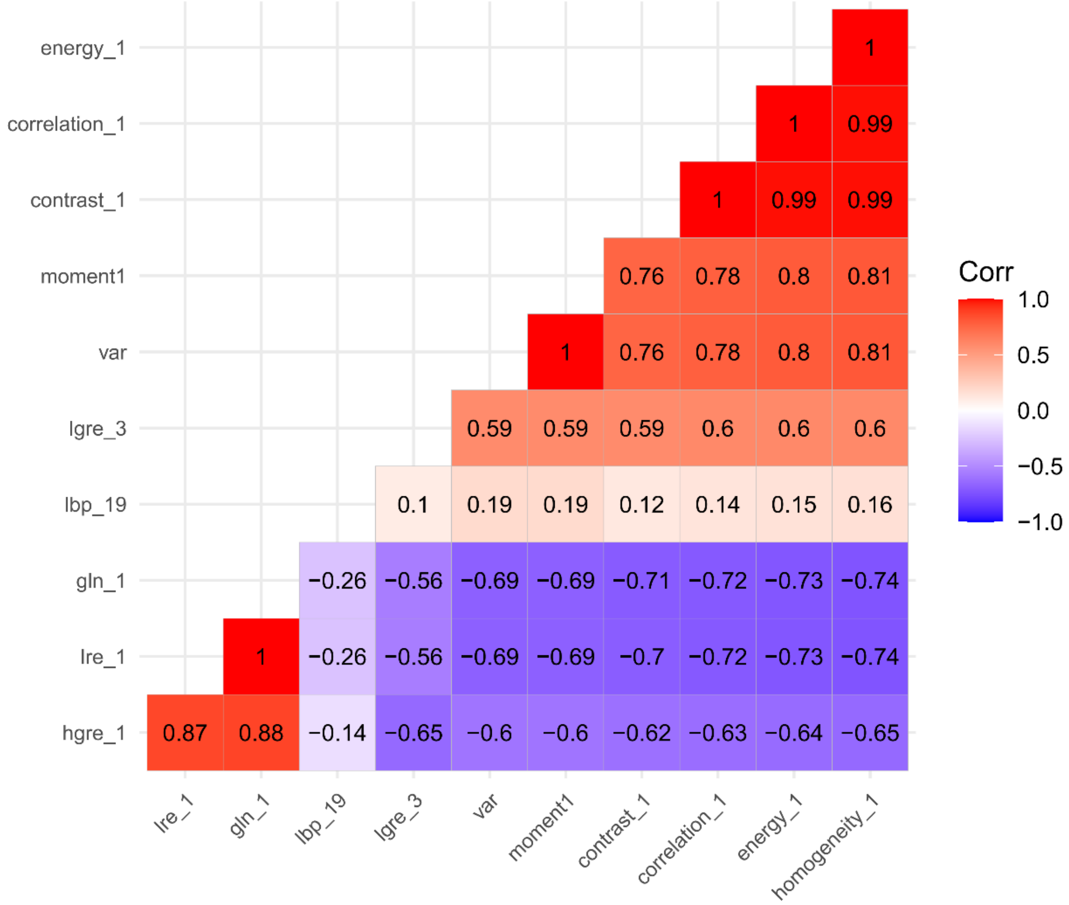

Supplementary Figure S1. The correlations among 11 selected features.

2.2 Supplemental Tables

Table S1 [4]. Overview of extracted features and methods of the feature extraction*.

Extracted features
Mean, variance, skewness, kurtosis, 5th to 10th central moments of the images (N =10).
Gray-level co-occurrence matrix (GLCM) features: Contrast, correlation, energy, homogeneity, angular second moment and variance of the GLCM with 10 offsets (N = 60) [5, 6].
Local binary pattern (LBP) feature: Rotation invariant image description with LBP histogram Fourier features with sample number = 8 and radius = 3 (N = 38) [7].
Perception-based features: coarseness, contrast, directionality, line-likeliness and roughness (N = 5) [8].
Gray level run length (GLRL) features: Short- and long-run emphasis, gray-level nonuniformity, run-length nonuniformity, run percentage, low and high gray-level run emphasis, short-run low and high gray-level emphasis, long-run low and high level emphasis of the GLRL matrix with 4 offsets (N= 44) [9].

*N, the numbers of features. We extracted 157 features from all 5 sets of the distinct texture descriptors cited from previous publications and the number of features in each set is 10, 60, 38, 5 and 44, respectively.

Table S2 The comparisons of in the risk factors for sudden cardiac death between male patients with HCM and female patients .

SCD risk factors*	Male（*n*=203）	Female（*n*=176）	*P*
ESC risk predictors			
Age(years)	47.2±15.1	50.2±17.6	0.08
Peak LVOT obstruction, (mmHg) †	27.0±32.2	36.5±35.5	0.006*
NSVT, *n*(%)	27	32	0.19
Family history of SCD, *n*(%)	28	22	0.71
History of Syncope, *n*(%)	28	42	0.011*
LA size(mm)	39.6±7.1	41.0±7.9	0.08
Maximum LV wall thickness(mm)	22.9±6.2	22.0±5.0	0.31
**CMR risk predictor**			
LGE%	8.7±9.2	7.8±7.4	0.32

*ESC, European society of Cardiology; LOVT, left ventricular outflow tract gradient; NSVT, non-sustained ventricular tachycardia; LGE, late gadolinium enhancement.

3. Supplementary references

1. Wright MN, Ziegler A (2017) ranger: A Fast Implementation of Random Forests for High Dimensional Data in C++ and R. Journal of Statistical Software DOI: 10.18637/jss. v077.i01.

2. Therneau T (2020). A Package for Survival Analysis in R. R package version 3.2-7, https://CRAN.R-project.org/package=survival.

3. Friedman J, Hastie T, Tibshirani R (2010). “Regularization Paths for Generalized Linear Models via Coordinate Descent.” Journal of Statistical Software, 33, 1–22.

4.Wang J, Yang F, Liu W, et al (2020) Radiomic Analysis of Native T1 Mapping Images Discriminates Between MYH7 and MYBPC3-Related Hypertrophic Cardiomyopathy. J Magn Reson Imaging DOI: 10.1002/jmri.27209.

5. Haralick RM, Shanmugam K, Dinstein I (1973) Textural features for image classification, IEEE transactions on systems. Man Cybern 3:610-621.

6. Haralick RM, Shapiro LG (1992) Computer and robot vision, Vol.1. Boston: Addison-Wesley Longman Publishing Co., Inc.; p 460.

7. Ahonen T, Matas J, He C, et al (2009) Rotation invariant image description with local binary pattern histogram Fourier features. Image analysis, 16th Scandinavian Conference, SCIA 2009, Oslo, Norway; June 15–18, Proceedings DBLP, 2009.

8. Bianconi F, Alvarez-Larrán A, Fernández A (2015) Discrimination between tumour epithelium and stroma via perception-based features. Neurocomputing 154:119-126.

9. Tang X (1998) Texture information in run-length matrices. IEEE Trans Image Process 7:1602-1609.
